# Refined histopathological predictors of *BRCA1* and *BRCA2*mutation status: a large-scale analysis of breast cancer characteristics from the BCAC, CIMBA, and ENIGMA consortia

**DOI:** 10.1186/s13058-014-0474-y

**Published:** 2014-12-23

**Authors:** Amanda B Spurdle, Fergus J Couch, Michael T Parsons, Lesley McGuffog, Daniel Barrowdale, Manjeet K Bolla, Qin Wang, Sue Healey, Rita Katharina Schmutzler, Barbara Wappenschmidt, Kerstin Rhiem, Eric Hahnen, Christoph Engel, Alfons Meindl, Nina Ditsch, Norbert Arnold, Hansjoerg Plendl, Dieter Niederacher, Christian Sutter, Shan Wang-Gohrke, Doris Steinemann, Sabine Preisler-Adams, Karin Kast, Raymonda Varon-Mateeva, Steve Ellis, Debra Frost, Radka Platte, Jo Perkins, D Gareth Evans, Louise Izatt, Ros Eeles, Julian Adlard, Rosemarie Davidson, Trevor Cole, Giulietta Scuvera, Siranoush Manoukian, Bernardo Bonanni, Frederique Mariette, Stefano Fortuzzi, Alessandra Viel, Barbara Pasini, Laura Papi, Liliana Varesco, Rosemary Balleine, Katherine L Nathanson, Susan M Domchek, Kenneth Offitt, Anna Jakubowska, Noralane Lindor, Mads Thomassen, Uffe Birk Jensen, Johanna Rantala, Åke Borg, Irene L Andrulis, Alexander Miron, Thomas VO Hansen, Trinidad Caldes, Susan L Neuhausen, Amanda E Toland, Heli Nevanlinna, Marco Montagna, Judy Garber, Andrew K Godwin, Ana Osorio, Rachel E Factor, Mary B Terry, Timothy R Rebbeck, Beth Y Karlan, Melissa Southey, Muhammad Usman Rashid, Nadine Tung, Paul DP Pharoah, Fiona M Blows, Alison M Dunning, Elena Provenzano, Per Hall, Kamila Czene, Marjanka K Schmidt, Annegien Broeks, Sten Cornelissen, Senno Verhoef, Peter A Fasching, Matthias W Beckmann, Arif B Ekici, Dennis J Slamon, Stig E Bojesen, Børge G Nordestgaard, Sune F Nielsen, Henrik Flyger, Jenny Chang-Claude, Dieter Flesch-Janys, Anja Rudolph, Petra Seibold, Kristiina Aittomäki, Taru A Muranen, Päivi Heikkilä, Carl Blomqvist, Jonine Figueroa, Stephen J Chanock, Louise Brinton, Jolanta Lissowska, Janet E Olson, Vernon S Pankratz, Esther M John, Alice S Whittemore, Dee W West, Ute Hamann, Diana Torres, Hans Ulrich Ulmer, Thomas Rüdiger, Peter Devilee, Robert AEM Tollenaar, Caroline Seynaeve, Christi J Van Asperen, Diana M Eccles, William J Tapper, Lorraine Durcan, Louise Jones, Julian Peto, Isabel dos-Santos-Silva, Olivia Fletcher, Nichola Johnson, Miriam Dwek, Ruth Swann, Anita L Bane, Gord Glendon, Anna M Mulligan, Graham G Giles, Roger L Milne, Laura Baglietto, Catriona McLean, Jane Carpenter, Christine Clarke, Rodney Scott, Hiltrud Brauch, Thomas Brüning, Yon-Dschun Ko, Angela Cox, Simon S Cross, Malcolm WR Reed, Jan Lubinski, Katarzyna Jaworska-Bieniek, Katarzyna Durda, Jacek Gronwald, Thilo Dörk, Natalia Bogdanova, Tjoung-Won Park-Simon, Peter Hillemanns, Christopher A Haiman, Brian E Henderson, Fredrick Schumacher, Loic Le Marchand, Barbara Burwinkel, Frederik Marme, Harald Surovy, Rongxi Yang, Hoda Anton-Culver, Argyrios Ziogas, Maartje J Hooning, J Margriet Collée, John WM Martens, Madeleine MA Tilanus-Linthorst, Hermann Brenner, Aida Karina Dieffenbach, Volke Arndt, Christa Stegmaier, Robert Winqvist, Katri Pylkäs, Arja Jukkola-Vuorinen, Mervi Grip, Annika Lindblom, Sara Margolin, Vijai Joseph, Mark Robson, Rohini Rau-Murthy, Anna González-Neira, José Ignacio Arias, Pilar Zamora, Javier Benítez, Arto Mannermaa, Vesa Kataja, Veli-Matti Kosma, Jaana M Hartikainen, Paolo Peterlongo, Daniela Zaffaroni, Monica Barile, Fabio Capra, Paolo Radice, Soo H Teo, Douglas F Easton, Antonis C Antoniou, Georgia Chenevix-Trench, David E Goldgar, EMBRACE Group, GENICA Network, HEBON Group, kConFab Investigators

**Affiliations:** 1 Department of Genetics and Computational Biology, QIMR Berghofer Medical Research Institute, 300 Herston Road, Brisbane, 4006 QLD Australia; 2 Department of Laboratory Medicine and Pathology, Mayo Clinic, 200 First Street SW, Rochester, 55905 MN USA; 3 Centre for Cancer Genetic Epidemiology, Department of Public Health and Primary Care, University of Cambridge, Cambridge, UK; 4 Centre for Cancer Genetic Epidemiology, Department of Oncology, University of Cambridge, Cambridge, UK; 5 Center for Hereditary Breast and Ovarian Cancer, Center for Integrated Oncology (CIO) and Center for Molecular Medicine Cologne (CMMC), Medical Faculty, University of Cologne and University Hospital Cologne, Cologne, Germany; 6 Institute for Medical Informatics, Statistics and Epidemiology, University of Leipzig, Härtelstrasse 16-18, Leipzig, 04107 Germany; 7 Division of Gynaecology and Obstetrics, Technische Universität München, Ismaninger Straße 22, Munich, 81675 Germany; 8 Department of Gynaecology and Obstetrics, Ludwig-Maximilians-Universität, Maistrasse 11, Munich, 80337 Germany; 9 Department of Gynaecology and Obstetrics, University Hospital of Schleswig-Holstein, Campus Kiel, Christian-Albrechts University Kiel, Kiel, Germany; 10 Institute of Human Genetics, University Hospital of Schleswig-Holstein, Campus Kiel, Christian-Albrechts University Kiel, Kiel, Germany; 11 University Medical Center Schleswig-Holstein, Kiel, Germany; 12 Department of Gynaecology and Obstetrics, University Hospital Düsseldorf, Heinrich-Heine University Düsseldorf, Düsseldorf, Germany; 13 Institute of Human Genetics, University Hospital Heidelberg, Im Neuenheimer Feld 366, Heidelberg, 69120 Germany; 14 Department of Gynaecology and Obstetrics, University Hospital Ulm, Albert-Einstein-Allee 23, Ulm, 89081 Germany; 15 Institute of Cell and Molecular Pathology, Hannover Medical School, Hannover, Germany; 16 Institute of Human Genetics, University of Münster, Münster, Germany; 17 Department of Gynecology and Obstetrics, University Hospital Carl Gustav Carus, Technische Universität Dresden, Dresden, Germany; 18 Institute of Human Genetics, Campus Virchov Klinikum, Charite Berlin, Germany; 19 Genetic Medicine, Manchester Academic Health Sciences Centre, Central Manchester University Hospitals NHS Foundation Trust, Manchester, UK; 20 Clinical Genetics, Guy’s and St. Thomas’ NHS Foundation Trust, London, UK; 21 Oncogenetics Team, The Institute of Cancer Research and Royal Marsden NHS Foundation Trust, Sutton, UK; 22 Yorkshire Regional Genetics Service, Chapel Allerton Hospital, Leeds, UK; 23 Department of Clinical Genetics, Southern General Hospital, 1345 Glovan Rd, Glasgow, G51 4TF UK; 24 West Midlands Regional Genetics Service, Birmingham Women’s Hospital Healthcare NHS Trust, Edgbaston, Birmingham UK; 25 Unit of Medical Genetics, Department of Preventive and Predictive Medicine, Fondazione IRCCS Istituto Nazionale dei Tumori (INT), Via Giacomo Venezian, 1, Milan, 20133 Italy; 26 Division of Cancer Prevention and Genetics, Istituto Europeo di Oncologia (IEO), Via Giuseppe Ripamonti, 435, Milan, 20141 Italy; 27 IFOM, Fondazione Istituto FIRC di Oncologia Molecolare, Via Adamello, 16, Milan, 20139 Italy; 28 Cogentech Cancer Genetic Test Laboratory, Via Adamello, 16, Milan, 20139 Italy; 29 Division of Experimental Oncology 1, CRO Aviano National Cancer Institute, Via Franco Gallini 2, Aviano, 33081 PN Italy; 30 Department of Medical Sciences, University of Turin, Via Santena 19, Turin, 10126 Italy; 31 AOU Città della Salute e della Scienza, corso Bramante 88, 10126 Turin Italy; 32 Unit of Medical Genetics, Department of Biomedical, Experimental and Clinical Sciences, University of Florence, Viale Pieraccini 6, Florence, 50139 Italy; 33 Unit of Hereditary Cancer, IRCCS AOU San Martino - IST Istituto Nazionale per la Ricerca sul Cancro, largo Rosanna Benzi 10, Genoa, 16132 Italy; 34 Western Sydney and Nepean Blue Mountains Local Health Districts, Westmead Millennium Institute for Medical Research, University of Sydney, 176 Hawkesbury Rd, Westmead, NSW 2145 Australia; 35 Abramson Cancer Center, University of Pennsylvania, 3400 Civic Center Boulevard, Philadelphia, 19104 PA USA; 36 Clinical Genetics Service, Department of Medicine, Memorial Sloan-Kettering Cancer Center, 417 East 68th Street, New York, 10021 NY USA; 37 Department of Genetics and Pathology, Pomeranian Medical University, Połabska 4, Szczecin, 70-115 Poland; 38 Department of Health Sciences Research, Mayo Clinic, 13400 E. Scottsdale Blvd., Scottsdale, AZ USA; 39 Department of Clinical Genetics, Odense University Hospital, Sonder Boulevard 29, Odense, C, Denmark; 40 Department of Clinical Genetics, Aarhus University Hospital, Brendstrupgaardsvej 21C, Aarhus, N, Denmark; 41 Department of Clinical Genetics, Karolinska University Hospital L5:03, Stockholm, S-171 76 Sweden; 42 Department of Oncology, Clinical Sciences, Lund University and Skåne University Hospital, Lund, Sweden; 43 Department of Molecular Genetics, University of Toronto, 1 King’s College Circle, Toronto, M5S 1A8 ON Canada; 44 Lunenfeld-Tanenbaum Research Institute of Mount Sinai Hospital, 600 University Avenue, Toronto, M5G 1X5 ON Canada; 45 Department of Genetics and Genome Services, Case Western Reserve University Medical School, 2109 Adelbert Rd, Cleveland, 44106-4955 OH USA; 46 Center for Genomic Medicine, Rigshospitalet, Copenhagen University Hospital, Blegdamsvej 9, Copenhagen, DK-2100 Denmark; 47 Molecular Oncology Laboratory, Hospital Clinico San Carlos, IdISSC, Martin Lagos s/n, Madrid, Spain; 48 Beckman Research Institute of City of Hope, 1500 East Duarte Rd, Duarte, 91010 CA USA; 49 Divison of Human Cancer Genetics, Departments of Internal Medicine and Molecular Virology, Immunology and Medical Genetics, Comprehensive Cancer Center, The Ohio State Universit, 998 Biomedical Research Tower, Columbus, OH USA; 50 Department of Obstetrics and Gynecology, University of Helsinki and Helsinki University Central Hospital, Haartmaninkatu 8, Helsinki, FI-00029 HUS Finland; 51 Immunology and Molecular Oncology Unit, Istituto Oncologico Veneto IOV - IRCCS, Via Gattamelata 64, Padua, Italy; 52 Dana-Farber Cancer Institute, 450 Brookline Avenue, Boston, MA USA; 53 Department of Pathology and Laboratory Medicine, University of Kansas Medical Center, 3901 Rainbow Boulevard,4019 Wahl Hall East, Kansas, MS 3040 KS USA; 54 Human Genetics Group, Human Cancer Genetics Program, Spanish National Cancer Research Centre (CNIO), C/Melchor Fernández, Almagro 3, Madrid, 28029 Spain; 55 Biomedical Network on Rare Diseases (CIBERER), Madrid, Spain; 56 Department of Pathology, University of Utah Health Sciences Center, 50 N Medical Dr, Salt Lake City, 84132 UT USA; 57 Department of Epidemiology, Columbia University, New York, NY USA; 58 Women’s Cancer Program at the Samuel Oschin Comprehensive Cancer Institute, Cedars-Sinai Medical Center, 8700 Beverly Boulevard, Suite 290 W, Los Angeles, CA USA; 59 Genetic Epidemiology Laboratory, Department of Pathology, University of Melbourne, Parkville, Victoria Australia; 60 Molecular Genetics of Breast Cancer, Deutsches Krebsforschungszentrum (DKFZ), Im Neuenheimer Feld 580, Heidelberg, 69120 Germany; 61 Department of Basic Sciences, Shaukat Khanum Memorial Cancer Hospital and Research Centre (SKMCH & RC) 7A, Block R3, Johar, Pakistan; 62 331 Brookline Avenue, Boston, 02215 MA USA; 63 Department of Public Health and Primary Care, University of Cambridge, Cambridge, UK; 64 Department of Oncology, University of Cambridge, Cambridge, UK; 65 Department of Medical Epidemiology and Biostatistics, Karolinska Institutet, Nobels väg 12A, Stockholm, SE-17177 Sweden; 66 Netherlands Cancer Institute, Antoni van Leeuwenhoek Hospital, Plesmanlaan 121, Amsterdam, 1066 CX Netherlands; 67 David Geffen School of Medicine, Department of Medicine Division of Hematology and Oncology, University of California at Los Angeles, 10833 Le Conte Avenue, Los Angeles, 90095 CA USA; 68 Department of Gynecology and Obstetrics, University Breast Center Franconia, University Hospital Erlangen, Friedrich-Alexander University Erlangen-Nuremberg, Comprehensive Cancer Center Erlangen-EMN, Universitätsstrasse 21-23, Erlangen, 91054 Germany; 69 Institute of Human Genetics, University Hospital Erlangen, Friedrich Alexander University Erlangen-Nuremberg, Schlossplatz 4, Erlangen, 91054 Germany; 70 Jonsson Comprehensive Cancer Center, University of California-Los Angeles, 10833 Le Conte Ave, Los Angeles, 90024 CA USA; 71 Copenhagen General Population Study, Herlev Hospital, Copenhagen University Hospital, Herlev Ringvej 74, Herlev, 2730 Denmark; 72 Department of Clinical Biochemistry, Herlev Hospital, Copenhagen University Hospital, Herlev Ringvej 74, Herlev, 2730 Denmark; 73 Faculty of Health and Medical Sciences, University of Copenhagen, Blegdamsvej 3B, Copenhagen N, 2200 Denmark; 74 Department of Breast Surgery, Herlev Hospital, Copenhagen University Hospital, Herlev Ringvej 74, Herlev, 2730 Denmark; 75 Division of Cancer Epidemiology, German Cancer Research Center (DKFZ), Im Neuenheimer Feld 280, Heidelberg, 69120 Germany; 76 Department of Cancer Epidemiology/Clinical Cancer Registry and Institute for Medical Biometrics and Epidemiology, University Clinic Hamburg-Eppendorf, Martinistraße 52, Hamburg, 20246 Germany; 77 Department of Clinical Genetics, University of Helsinki and Helsinki University Central Hospital, Haartmaninkatu 8, Helsinki, FI-00029 HUS Finland; 78 Department of Pathology, Helsinki University Central Hospital, Haartmaninkatu 8, Helsinki, FI-00029 HUS Finland; 79 Department of Oncology, University of Helsinki and Helsinki University Central Hospital, Haartmaninkatu 8, Helsinki, FI-00029 HUS, Finland; 80 Division of Cancer Epidemiology and Genetics, National Cancer Institute, 9609 Medical Center Drive, Rockville, 20850 MD USA; 81 Department of Cancer Epidemiology and Prevention, M. Sklodowska-Curie Memorial Cancer Center & Institute of Oncology, Warsaw, Poland; 82 Department of Health Sciences Research, Mayo Clinic, 200 First Street SW, Rochester, 55905 MN USA; 83 Cancer Prevention Institute of California, 2201 Walnut Avenue #300, Fremont, 94538 CA USA; 84 Department of Health Research and Policy, Stanford University School of Medicine, 291 Campus Drive, Stanford, 94305 CA USA; 85 Institute of Human Genetics, Pontificia University Javeriana, Carrera 7, Bogotá, 11001000 DC Colombia; 86 Frauenklinik der Stadtklinik Baden-Baden, Balger Straße 50, Baden-Baden, 76532 Germany; 87 Institute of Pathology, Städtisches Klinikum Karlsruhe, Moltkestraße 90, Karlsruhe, 76133 Germany; 88 Department of Human Genetics & Department of Pathology, Leiden University Medical Center, Einthovenweg 20, Leiden, 2333 ZC Netherlands; 89 Department of Surgical Oncology, Leiden University Medical Center, Einthovenweg 20, Leiden, 2333 ZC Netherlands; 90 Family Cancer Clinic, Department of Medical Oncology, Erasmus MC-Daniel den Hoed Cancer Centrer, Groene Hilledijk 301, EA Rotterdam, 3075 Netherlands; 91 Department of Clinical Genetics, Leiden University Medical Center, Einthovenweg 20, ZC Leiden, 2333 Netherlands; 92 Faculty of Medicine, University of Southampton, University Road, Southampton, SO17 1BJ England; 93 Queen Mary University London, Mile End Road, London, E1 4NS England; 94 Department of Non-Communicable Disease Epidemiology, London School of Hygiene and Tropical Medicine, Keppel Street, London, WC1E 7HT UK; 95 Breakthrough Breast Cancer Research Centre, The Institute of Cancer Research, 237 Fulham Road, London, SW3 6JB UK; 96 Department of Molecular and Applied Biosciences, Faculty of Science and Technology, University of Westminster, 115 New Cavendish Street, London, W1W 6UW UK; 97 Department of Pathology & Molecular Medicine, Juravinski Hospital, Concession St, Hamilton, L8V 1C3 Ontario Canada; 98 Cancer Centre, McMaster University, Hamilton, L8V 1C4 ON Canada; 99 Ontario Cancer Genetics Network, Lunenfeld-Tanenbaum Research Institute of Mount Sinai Hospital, 600 University Avenue, Toronto, M5G 1X5 ON Canada; 100 Department of Pathology, University Health Network, Toronto, M5G 2C4 ON Canada; 101 Department of Laboratory Medicine and Pathobiology, University of Toronto, 1 King’s College Circle, Toronto, M5S 1A8 ON Canada; 102 Cancer Epidemiology Centre, Cancer Council Victoria, 615 St Kilda Rd, Melbourne, 3004 Victoria Australia; 103 Centre for Epidemiology and Biostatistics, Melbourne School of Population and Global Health, The University of Melbourne, The University of Melbourne, Level 1, 723 Swanston Street, Melbourne, 3010 Victoria Australia; 104 Anatomical Pathology, The Alfred Hospital, 55 Commercial Rd, Melbourne, 3004 Victoria Australia; 105 Australian Breast Cancer Tissue Bank, Westmead Millennium Institute, University of Sydney, Darcy Rd, Sydney, NSW 2145 Australia; 106 Westmead Institute for Cancer Research, University of Sydney, Darcy Road, Sydney, NSW 2145 Australia; 107 Discipline of Medical Genetics, School of Biomedical Sciences and Pharmacy, Faculty of Health, University of Newcastle, University Drive, Callaghan, NSW 2305 Australia; 108 Division of Genetics, Hunter Area Pathology Service, John Hunter Hospital, Lookout Road, New Lambton Heights, Newcastle, NSW 2305 Australia; 109 University of Tübingen, Geschwister-Scholl-Platz, Tübingen, 72074 Germany; 110 Dr. Margarete Fischer-Bosch Institute of Clinical Pharmacology, Auerbachstraße 112, Stuttgart, 70376 Germany; 111 Institute for Prevention and Occupational Medicine of the German Social Accident Insurance (IPA), Bürkle-de-la-Camp-Platz 1, Bochum, 44789 Germany; 112 Evangelische Kliniken Bonn gGmbH, Johanniter Krankenhaus, Johanniterstraße 3, Bonn, 53113 Germany; 113 Department of Oncology, CRUK/YCR Sheffield Cancer Research Centre, University of Sheffield, Beech Hill Road, Sheffield, S10 2RX UK; 114 Academic Unit of Pathology, Department of Neuroscience, University of Sheffield, 385a Glossop Road, Sheffield, S10 2HQ UK; 115 Clinics of Radiation Oncology, Hannover Medical School, Carl-Neuberg-Straße 1, Hannover, 30625 Germany; 116 Department of Obstetrics and Gynaecology, Hannover Medical School, Carl-Neuberg-Straße 1, Hannover, 30625 Germany; 117 Department of Preventive Medicine, Keck School of Medicine, University of Southern California, Health Science Campus, 1975 San Pablo St., Los Angeles, 90033 CA USA; 118 Epidemiology Program, Cancer Research Center, University of Hawaii, 701 Ilalo Street, Honolulu, 96813 HI USA; 119 Department of Obstetrics and Gynecology, University of Heidelberg, Im Neuenheimer Feld 672, Heidelberg, 69120 Germany; 120 Molecular Epidemiology Group,, German Cancer Research Center (DKFZ), Im Neuenheimer Feld 280, Heidelberg, 69120 Germany; 121 National Center for Tumor Diseases, University of Heidelberg, Im Neuenheimer Feld 280, Heidelberg, 69120 Germany; 122 Department of Epidemiology, University of California Irvine, 224 Irvine Hall, Irvine, 92697 CA USA; 123 Department of Medical Oncology, Family Cancer Clinic, Erasmus University Medical Center, Rotterdam, 3008 AE Netherlands; 124 Department of Clinical Genetics, Family Cancer Clinic, Erasmus University Medical Center, Rotterdam, 3000 CA Netherlands; 125 Department of Surgical Oncology, Erasmus University Medical Center, Doctor Molewaterplein 50-60, Rotterdam, Netherlands; 126 Division of Clinical Epidemiology and Aging Research, German Cancer Research Center (DKFZ), Im Neuenheimer Feld 280, Heidelberg, 69120 Germany; 127 Saarland Cancer Registry, Präsident-Baltz-Straße 5, Saarbrücken, 66119 Germany; 128 Laboratory of Cancer Genetics and Tumor Biology, Department of Clinical Chemistry and Biocenter Oulu, University of Oulu, NordLab Oulu/Oulu University Hospital, Aapistie 5A, Oulu, FI-90220 Finland; 129 Department of Oncology, Oulu University Hospital, University of Oulu, Kajaanintie 50, Oulu, FI-90220 Finland; 130 Department of Surgery, Oulu University Hospital, University of Oulu, Kajaanintie 50, Oulu, FI-90220 Finland; 131 Department of Molecular Medicine and Surgery, Karolinska Institutet, Nobels väg 12A, Stockholm, SE-17177 Sweden; 132 Department of Oncology and Pathology, Karolinska Institutet, Nobels väg 12A, Stockholm, SE-17177 Sweden; 133 Human Genotyping-CEGEN Unit, Human Cancer Genetics Program, Spanish National Cancer Research Centre (CNIO), C/Melchor Fernández, Almagro 3, 28029 Madrid, Spain; 134 Servicio de Cirugía General y Especialidades, Hospital Monte Naranco, Av Doctores Fernández Vega, 107, Oviedo, 33012 Asturias, Spain; 135 Servicio de Oncología Médica, Hospital Universitario La Paz, Paseo de la Castellana, 261, Madrid, 28046 Spain; 136 Centro de Investigación en Red de Enfermedades Raras (CIBERER), Valencia, Spain; 137 School of Medicine, Institute of Clinical Medicine, Pathology and Forensic Medicine, University of Eastern Finland, Yliopistonranta 1C, Kuopio, FI-70211 Finland; 138 Imaging Center, Department of Clinical Pathology, Kuopio University Hospital, Puijonlaaksontie 2, Kuopio, 70210 Finland; 139 Cancer Center, Kuopio University Hospital, Puijonlaaksontie 2, Kuopio, 70210 Finland; 140 Unit of Molecular Bases of Genetic Risk and Genetic Testing, Department of Preventive and Predictive Medicine, Fondazione IRCCS Istituto Nazionale dei Tumori (INT), Via Giacomo Venezian 1, Milan, 20133 Italy; 141 Cancer Research Initiatives Foundation, Sime Darby Medical Centre, 1 Jalan SS 12/1a, Ss 12, Subang Jaya, Selangor Malaysia; 142 Faculty of Medicine, University Malaya Cancer Research Institute, University Malaya, Kuala Lumpur, 50603 Malaysia; 143 Department of Dermatology and Huntsman Cancer Institute, University of Utah School of Medicine, Salt Lake City, 84112 UT USA

## Abstract

**Introduction:**

The distribution of histopathological features of invasive breast tumors in *BRCA1* or *BRCA2* germline mutation carriers differs from that of individuals with no known mutation. Histopathological features thus have utility for mutation prediction, including statistical modeling to assess pathogenicity of *BRCA1* or *BRCA2* variants of uncertain clinical significance. We analyzed large pathology datasets accrued by the Consortium of Investigators of Modifiers of *BRCA1*/2 (CIMBA) and the Breast Cancer Association Consortium (BCAC) to reassess histopathological predictors of *BRCA1* and *BRCA2* mutation status, and provide robust likelihood ratio (LR) estimates for statistical modeling.

**Methods:**

Selection criteria for study/center inclusion were estrogen receptor (ER) status or grade data available for invasive breast cancer diagnosed younger than 70 years. The dataset included 4,477 *BRCA1* mutation carriers, 2,565 *BRCA2* mutation carriers, and 47,565 BCAC breast cancer cases. Country-stratified estimates of the likelihood of mutation status by histopathological markers were derived using a Mantel-Haenszel approach.

**Results:**

ER-positive phenotype negatively predicted *BRCA1* mutation status, irrespective of grade (LRs from 0.08 to 0.90). ER-negative grade 3 histopathology was more predictive of positive *BRCA1* mutation status in women 50 years or older (LR = 4.13 (3.70 to 4.62)) versus younger than 50 years (LR = 3.16 (2.96 to 3.37)). For *BRCA2*, ER-positive grade 3 phenotype modestly predicted positive mutation status irrespective of age (LR = 1.7-fold), whereas ER-negative grade 3 features modestly predicted positive mutation status at 50 years or older (LR = 1.54 (1.27 to 1.88)). Triple-negative tumor status was highly predictive of *BRCA1* mutation status for women younger than 50 years (LR = 3.73 (3.43 to 4.05)) and 50 years or older (LR = 4.41 (3.86 to 5.04)), and modestly predictive of positive *BRCA2* mutation status in women 50 years or older (LR = 1.79 (1.42 to 2.24)).

**Conclusions:**

These results refine likelihood-ratio estimates for predicting *BRCA1* and *BRCA2* mutation status by using commonly measured histopathological features. Age at diagnosis is an important variable for most analyses, and grade is more informative than ER status for *BRCA2* mutation carrier prediction. The estimates will improve *BRCA1* and *BRCA2* variant classification and inform patient mutation testing and clinical management.

**Electronic supplementary material:**

The online version of this article (doi:10.1186/s13058-014-0474-y) contains supplementary material, which is available to authorized users.

## Introduction

It is well established that *BRCA1*-related breast tumors, as a group, differ from non-*BRCA1* tumors in terms of histological phenotype. Tumors of *BRCA1* mutation carriers are more likely to be high-grade with medullary subtype features, including greatly increased mitotic count, pushing margins, lymphocytic infiltrate, trabecular growth pattern, and necrosis [1]-[3]. Consistent with overrepresentation of a basal phenotype, a number of immunohistochemical (IHC) markers have been shown to be of value in assessing *BRCA1* tumor phenotype in female patients, including estrogen receptor (ER), progesterone receptor (PR), human Epidermal Growth Factor Receptor 2 (HER2), p53, cytokeratin 5/6 (CK5/6), cytokeratin 14 (CK14), cytokeratin 17 (CK17), and epidermal growth factor receptor (EGFR) [4]-[8]. In addition, several studies reported that reduced expression of CK8/18 can discriminate the basal tumors of *BRCA1* mutation carriers from basal tumors of noncarriers [9],[10], whereas loss of phosphatase and tensin homolog (PTEN), together with triple-negative (TN; ER-, PR-, HER2-) status, was reported to improve the sensitivity of *BRCA1* mutation prediction in a study of Asian breast cancer patients [11]. The introduction of PTEN to *BRCA1* mutation-prediction algorithms is supported by single-cell analyses of temporal somatic events in *BRCA1* breast tumor tissue, which revealed that loss of PTEN is an early event in the development of *BRCA1* basal-like tumors, whereas *TP53* mutations occur first in most luminal *BRCA1* tumors [12].

The breast tumor phenotype of female *BRCA2* female mutation carriers is less distinctive than that of *BRCA1* mutation carriers [1],[13],[14]. Nevertheless, reports based on IHC or expression array analysis have shown that *BRCA2* breast tumors are predominantly of the luminal B subtype [13],[15], and are more likely than non-*BRCA2* tumors to be ER positive and high grade, with reduced tubule formation and continuous pushing margins [2],[13].

A number of these histopathological features have been incorporated into prediction models or have been proposed as selection criteria for prioritizing testing of breast cancer patients for *BRCA1* and *BRCA2* mutations [11],[16]-[24]. These findings have also served as the basis for including independently predictive tumor histopathological features as a component of the multifactorial likelihood model for clinical classification of *BRCA1/2* variants of uncertain significance [25]. The current iteration of the model includes likelihood ratio (LR) estimates of pathogenicity for combined ER and grade or combined ER, CK5/6, and CK14 status, for analysis of *BRCA1* variants, and tubule formation for *BRCA2* [26]-[29]. However, these LR estimates were derived from analyses of relatively small datasets including a maximum of 600 mutation carriers and 288 noncarriers [4],[6], and have not been directly validated.

We conducted analyses of large pathology datasets accrued by the Consortium of Investigators of Modifiers of *BRCA1/2* (CIMBA) and the Breast Cancer Association Consortium (BCAC) to reassess previously reported histopathological predictors of *BRCA1* and *BRCA2* mutation status. The results provide more-refined LR estimates for downstream multifactorial likelihood analysis and for prediction of *BRCA1* and *BRCA2* mutation status.

## Methods

### Access to data and ethics approvals

*ENIGMA* (Evidence-based Network for the Interpretation of Germline Mutant Alleles) is a research consortium aimed to improve methods to assess the clinical significance in breast cancer susceptibility genes [30]. Considerable overlap in membership exists between ENIGMA, CIMBA, and BCAC. As a collaboration between the three consortia, investigators in ENIGMA accessed CIMBA and BCAC datasets for approved pathology-related analyses relevant to the purposes of ENIGMA. The collection of clinical, pathology, and genetic data by CIMBA and BCAC has been previously approved for ongoing research studies by the local ethics committee relevant to each of the participating CIMBA and BCAC studies, and all participants provided informed consent to the relevant participating CIMBA and BCAC sites for such ongoing studies.

Research analyses specific to this study were carried out using only de-identified data, with approval from the Human Research Ethics Committee of the QIMR Berghofer Medical Research Institute, and the Institutional Review Board of the University of Utah.

### Sample sets

#### CIMBA

The Consortium of Investigators of Modifiers of *BRCA1/2* (CIMBA; [31]) is a consortium established to conduct large-scale research studies of carriers of germline *BRCA1* or *BRCA2* pathogenic mutations [32]. Specifically, carriers of variants of uncertain significance are ineligible for entry into CIMBA. The major focus is discovery and validation of genetic factors that modify risk of breast and ovarian cancer in *BRCA1* and *BRCA2* mutation carriers, with consideration of risk stratified by tumor histologic features. Contributing centers provide information relevant to analyses, including year of birth, age at diagnosis of breast and/or ovarian cancer, cancer behavior (invasive, *in situ*), basic histology, and other pathology measures for breast and ovarian tumors from study participants. Pathology information is extracted mainly from pathology reports, although a small subset of contributing centers have conducted centralized pathology review and/or supplemented clinical IHC results with research testing of tumor material (for example, 5% of ER pathology results were centrally reviewed) [33]. All CIMBA centers with ER and grade data available in the CIMBA database that were from countries with pathology data available from population (presumed noncarrier) reference cases in BCAC (see later) were included in the analyses. Variables included were as follows: gene mutated, mutation nomenclature (and mutation type, for example, truncating, missense, and so on), date of birth, age and date of diagnosis of breast cancer(s), breast cancer behavior, ER status, PR status, HER2 status, Cytokeratin 5 or 5/6 status, and grade. No CK14 IHC results were available. No dual-mutation carriers were found. Only invasive breast cancer cases diagnosed before age 70 years were included, to reduce the likelihood of phenocopy tumors not directly related to mutation status. Samples were included irrespective of ovarian cancer diagnoses. For individuals with two breast cancers (20% of cases), the breast cancer diagnosed closest in time to the entry into the CIMBA cohort was included preferentially.

#### BCAC

The Breast Cancer Association Consortium (BCAC [34]) was established to discover and validate genetic factors associated with risk of breast cancer in the general population [35]. BCAC also studies risk factors associated with tumor subtypes and tumor histologic features, and pathology data from participating centers are derived from pathology reports or center-specific research efforts. BCAC pathology data were checked and cleaned centrally [36]. BCAC centers were selected for inclusion in this analysis based on availability of ER and grade data. Studies in BCAC in which cases were ascertained on the basis of tumor characteristics (for example, the TN consortium) were excluded. Variables provided for analyses were as follows: study type (to identify within-study strata, and/or to define cohorts with familial cases), age at diagnosis of breast cancer(s), breast cancer behavior, ER status, PR status, HER2 status, CK5 or 5/6 status, and grade. No CK14 IHC results were available. The study design was noted as selected (familial and/or age-selected, relevant for 13 studies) or unselected (from population-based or hospital-based design), based on study-ascertainment criteria provided by the principal investigators of individual BCAC sites.

*BRCA1* and *BRCA2* germline mutation testing results were provided by 13 of the 36 BCAC studies (comprising 12% of BCAC individuals overall), nine of which used age/family history selection criteria for case ascertainment (with testing for 4% to 100% of these nine studies). The 345 known mutation carriers (189 *BRCA1*, 156 *BRCA2*) identified in BCAC were excluded. Analysis included subjects known to be noncarriers or untested for *BRCA1/2* mutations, with relevant pathology information for primary invasive breast cancer diagnosis younger than age 70 years. As for CIMBA, for individuals with two breast cancers (only 5% of all BCAC cases considered), the breast cancer diagnosed closest in time to the entry into the cohort was included preferentially.

### Statistical analysis

ER or grade data were available for 4,477 *BRCA1* mutation carriers, 2,565 *BRCA2* mutation carriers, and 47,565 BCAC breast cancer cases with no known mutation in *BRCA1* or *BRCA2* (presumed noncarriers). The numbers of subjects by country are shown in Table 1. Only countries with ≥200 cases in BCAC and ≥100 carriers in CIMBA were included in analyses to minimize potential bias due to country-specific patterns of pathology assessment. ER-negative, PR-negative, and HER2-negative tumors were categorized as triple-negative (TN). All other combinations of known ER, PR, and HER2 status for a single breast tumor were categorized as “Not TN.” CIMBA and BCAC studies contributing pathology data are noted in Additional file 1: Table S1. Final sample sizes for analyses are reported in footnotes to Tables 2 and 3, and Additional file 1, Tables S2 to S4.

**Table 1:** **Subjects in CIMBA and BCAC datasets with breast tumor ER or grade status, by country**

Country	CIMBA		BCAC
	Number *BRCA1*	Number *BRCA2*	Number BCAC noncarriers
Australia	363	293	2,014
Canada	97	57	927
Denmark	201	151	2,318
Finland	55	64	2,607
Germany	982	493	9,503
Italy	547	362	270
Netherlands	113	32	4,181
Poland	247	0	2,527
Spain	91	102	358
Sweden	158	34	5,266
United Kingdom	642	388	12,989
USA	981	589	4,605
***Total***	***4,477***	***2,565***	***47,565***

Data for primary breast tumor. ER, breast tumor estrogen receptor status; BCAC, Breast Cancer Association Consortium; CIMBA, Consortium for Investigator of Modifiers of *BRCA1* and *BRCA2*.

ER status was missing for 548 (12.2%) *BRCA1* carriers, 292 (11.4%) *BRCA2* carriers and 4,942 (10.4%) presumed noncarriers. Histological grade was missing for 890 (19.9%) *BRCA1* carriers, 555 (21.6%) *BRCA2* carriers, and 6,020 (12.7%) presumed noncarriers.

**Table 2:** **Estimated likelihood ratios for predicting** ***BRCA1*** **or** ***BRCA2*** mutation status defined by breast tumor ER and/or grade phenotype*

Gene	ER status	Grade	Diagnosis <50 years				Diagnosis ≥50-70 years			
			% Carriers (CIMBA)	% BCAC	LR	(95% CI)	% Carriers (CIMBA)	% BCAC	LR	(95% CI)
*BRCA1*	ER negative	Grade 1	0.8	1.4	0.59	(0.36-0.98)	0.6	1.2	0.51	(0.18-1.40)
	ER negative	Grade 2	9.8	6.7	1.36	(1.18-1.58)	13.3	6.1	2.34	(1.88-2.91)
	ER negative	Grade 3	67.1	20.8	3.16	(2.96-3.37)	54.5	12.8	4.13	(3.70-4.62)
	ER positive	Grade 1	1.0	13.7	0.08	(0.05-0.12)	2.3	20.6	0.11	(0.07-0.18)
	ER positive	Grade 2	7.4	36.1	0.21	(0.18-0.24)	14.6	43.6	0.34	(0.28-0.42)
	ER positive	Grade 3	13.9	21.2	0.64	(0.57-0.72)	14.7	15.8	0.90	(0.73-1.10)
			100%	100%			100%	100%		
	-	Grade 1	2.1	15.8	0.13	(0.10-0.16)	2.9	22.3	0.12	(0.08-0.18)
		Grade 2	18.1	42.8	0.38	(0.34-0.42)	28.7	49.1	0.57	(0.50-0.65)
		Grade 3	79.8	41.4	1.67	(1.62-1.78)	68.4	28.6	2.20	(2.01-2.71)
			100%	100%			100%	100%		
	ER negative	-	77.5	28.8	2.60	(2.47-2.73)	69.4	19.9	3.31	(3.03-3.61)
	ER positive		22.5	71.2	0.32	(0.29-0.34)	30.6	80.1	0.37	(0.32-0.42)
			100%	100%			100%	100%		
*BRCA2*	ER negative	Grade 1	0.7	1.4	0.51	(0.25-1.05)	1	1.2	0.86	(0.36-2.08)
	ER negative	Grade 2	3.4	6.7	0.49	(0.36-0.68)	5.2	6.1	0.89	(0.60-1.32)
	ER negative	Grade 3	14.6	20.8	0.69	(0.59-0.80)	20.8	12.8	1.54	(1.27-1.88)
	ER positive	Grade 1	4.9	13.7	0.37	(0.28-0.48)	6.6	20.6	0.32	(0.22-0.45)
	ER positive	Grade 2	37.8	36.1	1.07	(0.97-1.17)	37.5	43.6	0.89	(0.77-1.02)
	ER positive	Grade 3	38.7	21.2	1.77	(1.60-1.95)	28.9	15.8	1.76	(1.49-2.08)
			100%	100%			100%	100%		
	-	Grade 1	5.7	15.8	0.33	(0.26-0.41)	8.6	22.3	0.35	(0.27-0.46)
		Grade 2	41.8	42.8	0.88	(0.80-0.95)	42.0	49.1	0.81	(0.72-0.92)
		Grade 3	52.5	41.4	1.08	(1.00-1.17)	49.4	28.6	1.52	(1.35-1.71)
			100%	100%			100%	100%		
	ER negative	-	19.2	28.8	0.66	(0.59-0.74)	25.1	19.9	1.18	(1.01-1.38)
	ER positive		80.8	71.2	1.15	(1.08-1.22)	74.9	80.1	0.90	(0.82-0.98)
			100%	100%			100%	100%		

*Analyses stratified by country, as detailed in the methods section. LR, Likelihood ratio; ER, breast tumor estrogen-receptor status; BCAC, Breast Cancer Association Consortium, No known mutation status.

ER-Grade analysis included tumor phenotypes from 3,039 *BRCA1* mutation carriers (2,393 < 50 years at diagnosis, 646 ≥ 50 years), 1,718 *BRCA2* mutation carriers (1,217 < 50 years at diagnosis, 501 ≥ 50 years) and 36,603 BCAC cases with no report of positive *BRCA1/2* mutation status (12,584 < 50 years at diagnosis, 24,019 ≥ 50 years). Grade analysis included tumor phenotypes from 3,587 *BRCA1* mutation carriers (2,825 < 50 years at diagnosis, 762 ≥ 50 years), 2,010 *BRCA2* mutation carriers (1,415 < 50 years at diagnosis, 595 ≥ 50 years) and 41,545 BCAC cases with no report of positive *BRCA1/2* mutation status (14,678 < 50 years at diagnosis). ER analysis included tumor phenotypes from 3,929 *BRCA1* mutation carriers (3,106 < 50 years at diagnosis, 824 ≥ 50 years), 2,273 *BRCA2* mutation carriers (1,616 < 50 years at diagnosis, 657 ≥ 50 years), and 42,623 BCAC cases with no report of positive *BRCA1/2* mutation status (14,484 < 50 years at diagnosis, 28,139 ≥ 50 years). Percentages may not total 100 because of rounding error.

**Table 3:** **Estimated likelihood ratios for predicting** ***BRCA1*** **or** ***BRCA2*** **mutation status defined by breast tumor triple-negative phenotype**

Gene	Breast tumor phenotype	Diagnosis <50 years				Diagnosis ≥50 to 70 years			
		% Carriers	% BCAC	LR	(95% CI)	% Carriers	% BCAC	LR	(95% CI)
*BRCA1*	Triple-negative	67.3	17.5	3.73	(3.43-4.05)	57.7	12.9	4.41	(3.86-5.04)
	Not triple-negative	32.7	82.5	0.40	(0.37-0.44)	42.3	87.1	0.49	(0.42-0.56)
		100%	100%			100%	100%		
*BRCA2*	Triple-negative	13.0	17.5	0.72	(0.59-0.87)	23.5	12.9	1.79	(1.42-2.24)
	Not triple-negative	87.0	82.5	1.06	(0.98-1.15)	76.5	87.1	0.88	(0.78-1.00)
		100%	100%			100%	100%		

Analyses stratified by country, as described in the Methods section. Analysis included tumor phenotypes from 2,249 *BRCA1* mutation carriers (1,788 < 50 years, 461 ≥ 50 years), 1,195 *BRCA2* mutation carriers (859 < 50 years, 336 ≥ 50 years) and 19,178 BCAC cases with no report of positive *BRCA1/2* mutation status (7,103 < 50 years, 12,075 ≥ 50 years). LR, likelihood ratio. Triple-negative phenotype defined as ER-negative, PR-negative, HER2-negative; not triple-negative; all other combinations, with status measured for all three markers.

CK5/6 IHC data were available for only 128 *BRCA1* carriers, 78 *BRCA2* carriers and 6,796 BCAC cases with valid data on ER status. Numbers of carriers reduced further after country-matching, and frequencies differed significantly between countries for carriers. Cytokeratin analyses were thus not pursued further.

All statistical analyses were performed by using STATA version 12 (StatCorp, College Station, TX, USA). Statistical significance was defined as *P* <0.05.

We first examined whether family history was related to the predictor variables of interest in the BCAC sample set. Family-history information, defined as first-degree relative with breast cancer, was available for 30,223 individuals (7,547 reporting a family history of breast cancer). Logistic regression analyses were performed to predict ER status, grade 3, or TN status as a function of family history (defined as first-degree relative with breast cancer), adjusting for age at diagnosis and country. No significant effect was observed for family history on any of these histopathologic features, so we did not consider family history further in any analyses.

To identify the most important predictors of mutation status to be used in estimation of the likelihood ratios for classification of variants, we undertook a series of logistic regression analyses. These analyses compared *BRCA1* and *BRCA2* with the BCAC set. A sequential series of models with country and age (younger than 50 years versus 50 years or older) as a starting point and then adding ER, grade, and the ER/grade combination to test for interaction between ER and grade. For those cases who had data on TN status, we examined ER, ER and grade, ER TN, grade TN, and last, models with ER, grade, and TN. Likelihood ratio tests were used to determine the most parsimonious models for each gene.

We then estimated simple likelihood ratios of the form L[path| BRCA_i_/L[path|BRCA_0_], where i = 1, 2 to denote tumors from women with germline *BRCA1* and *BRCA2* mutations, respectively, and BRCA_0_ to denote cancers from women presumed to be without such mutations. For example, if *m BRCA1* tumors have a given histopathological feature of a total of *M* total carriers with measured data on this feature, and *s* noncarriers (in this case, from the BCAC set) of a total of S have the feature of interest, then the LR is estimated by (*m*/*M*)/(*s*/*S*). An approximate variance of log(LR) is given by Var(ln(LR) = [1/*m* – 1/*M* +1/*s* – 1/*S*]. Thus assuming a normal distribution for log(LR), 95% confidence limits are given by exp[ln(LR) ± 1.96√(Var(ln(LR))].

However, to account for potential differences between countries in the distributions of ER status and grade together with large differences in the ratio of carriers to noncarriers, we derived stratified estimates of LR by using a Mantel-Haenszel approach [37] with approximate 95% confidence intervals calculated according to Greenland and Robins [38]. The country-based strata considered were as follows: Australia, United Kingdom, Germany, USA, and all other countries (with smaller individual sample sizes) pooled. Both stratified and unstratified analyses were conducted for all ages, and by age at diagnosis younger than 50 years versus 50 years or older, when sufficient sample size was available in all groups.

We performed a series of sensitivity analyses to assess how the lack of *BRCA1/2* testing in the vast majority of BCAC cases might affect the Likelihood Ratio estimates reported here. First, we determined the probability that each untested BCAC case was a true noncarrier as follows: We calculated the probability, *a priori*, that each untested BCAC case carried a pathogenic *BRCA1* and/or *BRCA2* mutation by using the age-specific relative risks in Antoniou *et al.* [39] and assuming allele frequencies of pathogenic mutations in each gene of 0.0005. Next we calculated crude LRs for ER-negative and ER-positive tumor status by using only the ~6,000 BCAC cases that tested negative for *BRCA1/2*, and all the *BRCA1* and *BRCA2* carriers. Then, assuming the prior calculated in step 1, we used these preliminary ER LRs to calculate the posterior probability that each untested BCAC case had a mutation in *BRCA1* or *BRCA2* based on their ER status. We calculated the probability that each BCAC case was a true noncarrier for a mutation in either gene, as 1, brca1 probability minus brca2 probability.

Second we reestimated a subset of the LRs by using iterative sampling of BCAC cases from the posterior distribution calculated, as described. We generated a uniform random number for each case, and used this and the posterior probabilities to determine whether each of the untested BCAC cases was a noncarrier, a *BRCA1* carrier, or a *BRCA2* carrier. We then used these simulated data to reestimate LRs from the whole data set, adjusting for country, as in the initial analysis.

Further, to examine the effects of changes in pathology over time, potential racial/ethnic differences in these features, and possible survival bias, we performed three additional analyses, one estimating overall unstratified ER/grade LRs for diagnosis after 1989; one restricted to white European ancestry cases only; and another of only cases diagnosed within 5 years of recruitment (to avoid possible bias between tumor phenotype and survival).

## Results

The principal aim of this study was to reassess histopathological predictors of *BRCA1* and *BRCA2* mutation status by analysis of datasets considerably larger than those analyzed previously for this purpose, to provide more robust pathology-based likelihood ratios for use in assessing the pathogenicity of *BRCA1* or *BRCA2* variants. Our main analyses of breast tumor features included up to 3,929 *BRCA1* mutation carriers, 2,273 *BRCA2* mutation carriers, and 42,623 assumed *BRCA1* and *BRCA2* mutation-negative breast cancer cases (Tables 2 and 3). This large sample set allowed us to explore ER alone, grade alone, combined ER and grade stratified by age, and ER/PR/HER2 TN status as predictors of *BRCA1* and *BRCA2* mutation status.

### Logistic regression determining best histopathology predictors of mutation status

For *BRCA1* carriers, likelihood ratio tests indicated that both ER and grade were strong independent predictors of *BRCA1* status compared with the BCAC set (*P* <10^-20^). Marginal evidence suggested that considering grade and ER status jointly improved the fit compared with including them separately in the model (χ^2^ = 6.25, 2 *df*, *P* = 0.04). When we considered only cases in which TN status and grade were available, TN significantly added to the model fit, even with ER status in the model; the most parsimonious model included ER, grade, and TN status, which was significantly better than any model with only two of these included (χ^2^ = 83.8, 1 *df*, *P* <10^-20^). For *BRCA2* both ER and grade were highly significant predictors of mutation status, and the interaction of ER and grade was also quite significant (χ^2^ = 28.3, 2 *df*, *P* <10^-6^). The addition of TN did not improve the model fit significantly (*P* = 0.14) when ER and grade were included in the model. We thus considered ER, grade, and TN status in deriving likelihood ratio estimates for BRCA-mutation status.

### ER and grade as predictors of mutation status

The estimated likelihood ratios for predicting *BRCA1* or *BRCA2* mutation status defined by breast tumor ER-grade phenotype, adjusted for country by using stratified analysis, are shown in Table 2. Results based on pooled data unstratified for country, including cell counts, are shown in Additional file 1: Table S2. In general, the Mantel-Haenszel stratified LR estimates were quite similar to the pooled estimates, with stratified estimates most often closer to 1.0 (although not always). Significant between-country heterogeneity for the estimated likelihood ratios was most often observed with grade rather than ER or TN status. ER-positive cases were less likely to be carriers of a *BRCA1* mutation, irrespective of grade. Conversely, ER-negative cases with high-grade tumors were more likely to be *BRCA1* mutation carriers. Further, our analyses showed that ER-positive grade 3 tumors were modestly predictive of positive *BRCA2* mutation status (Table 2). The association of *BRCA2* mutation status with ER-positive high-grade tumors was not substantially different for women diagnosed at younger or older than age 50 years (LR <50 years = 1.77 (95% CI, 1.60 to 1.95), LR ≥50 years = 1.76 (95% CI, 1.49 to 2.08)). However, ER-negative grade 3 tumor status was modestly predictive of positive *BRCA2* mutation status in women diagnosed at 50 years or older (LR, 1.54; 95% CI = 1.27 to 1.88).

It is well known that ER and grade status are correlated, with ER-negative tumors more likely to present with high grade. Consistent with this, relatively few cases appeared in any of the sample sets with ER-negative grade 1 tumors. However, we estimated LRs for ER alone and grade alone to allow inclusion of pathology data in models for predicting *BRCA1* and *BRCA2* mutation status, in instances in which information for only one of these variables is available (Table 2). For example, for a woman diagnosed with breast cancer at 50 years or older, the LR in favor of positive *BRCA1* mutation status would be 3.5 if her tumor were known to be ER negative but grade status was unknown, and 2.4 if reported as grade 3 without information on ER status.

An acknowledged caveat to the inclusion of pathology data in multifactorial likelihood modeling is the underlying assumption that missense and in-frame deletions considered to be pathogenic mutations will exhibit the same tumor histopathological characteristics as do truncating mutations. The dataset in this study included 398 known pathogenic *BRCA1* missense mutation carriers (mainly C61G), and 44 pathogenic *BRCA2* missense mutation carriers with information on ER status or grade. Comparing the missense variants with the truncating set of mutations, we found no significant association of *BRCA1* mutation type with ER status (OR = 0.9; 95% CI, 0.7 to 1.2; *P* =0.4) or grade (OR = 1.15; 95% CI, 0.9 to 1.4; *P* =0.2) or *BRCA2* (OR = 2.7; CI, 0.9 to 7.6; =0.07 for ER; OR = 0.6 0.3 –to 1.2; *P* =0.14 for grade), although power was quite limited for *BRCA2*.

### Triple-negative (TN) phenotype in *BRCA1* and *BRCA2*carriers

Secondary country-stratified analysis of 2,249 *BRCA1*, 1,195 *BRCA2* and 19,178 assumed mutation-negative breast cancer cases (Table 3) indicated that TN tumor status is highly predictive of *BRCA1* mutation status for women diagnosed at younger than 50 years (LR = 3.73; 95% CI, 3.43 to 4.05) and at age 50 years or older (LR = 4.41; 95% CI 3.86 to 5.04), and results were little different for unstratified analysis (see Additional file 1: Table S3, also displaying cell counts).

Results also indicated that TN phenotype is modestly predictive of *BRCA2* mutation status in cases diagnosed at age 50 years or older (LR, 1.79; 95% CI = 1.42 to 2.24). This observation is explained by the lower frequency of the TN phenotype in noncarriers (12.9% 50 years or older) versus *BRCA2* mutation carriers (23.5% 50 years or older). Additional analysis considering grade and TN status combined (see Additional file 1: Table S4) did not show substantial improvement over LRs estimated for ER and grade combined (Table 2) or TN status (Table 3), although numbers in some cells were limited.

### Sensitivity analyses

With respect to the possible consequences of contamination by missed mutation carriers in the BCAC sample set, we first estimated which BCAC-untested cases were more likely to be an undetected mutation carrier, and then re-estimated a subset of the LRs by using iterative sampling of the control dataset. Based on age-specific relative risks, we estimated that there could be at most 796 *BRCA1* (1.7%) and 433 *BRCA2* (0.9%) undetected carriers in the reference dataset of 47,565 BCAC cases. Based on age and crude ER, LR estimated from true non-carriers in BCAC, of 41,515 BCAC cases whose genetic status was unknown, 34,869 (84%) had posterior probabilities of being a true *BRCA1/2*-negative case greater than 0.95, with the minimum posterior probability being 0.89. Repeating this sampling process a total of 5 times, the number of *BRCA1* carriers within the BCAC set ranged from 688 to 784, and the number of *BRCA2* carriers ranged from 410 to 455 (total carriers, 1,114 to 1,194). Re-estimation of a subset of LRs indicated that the LRs assuming all BCAC cases do not carry a pathogenic *BRCA1* or *BRCA2* mutation is quite close to what we would expect, had all individuals been tested. For ER-negative Grade 3 cases diagnosed at younger than 50 years, the original LR for *BRCA1* mutation status, assuming all BCAC cases were non-carriers, was 3.16, whereas the five replicates from iterative analysis ranged from 3.22 to 3.25. For TN tumor phenotype, the original LR for *BRCA1* mutation status was 3.73, whereas the median of the five replicates was 3.76.

In additional sensitivity analyses, we recalculated unstratified LRs for ER and grade combined, restricting the analyses to the subset of 36,522 (33,260 BCAC, 3,252 CIMBA) breast cancer cases of European ancestry, of which 31,374 (28,364 BCAC, 3,010 CIMBA) were diagnosed within 5 years of interview, and 40,874 (36,414 BCAC, 4,460 CIMBA) were diagnosed after 1989. Results were similar to those from the overall analyses, with LR estimates consistently within the confidence intervals of the overall analyses.

## Discussion

### Histopathological predictors of mutation status

This study assessing histopathological predictors of *BRCA1* and *BRCA2* mutation status is based on the largest sample set reported to date, and so provides more-precise estimates that account for age at diagnosis as a potential confounder. We also provide age-stratified LRs for ER alone and grade alone, which, although not as predictive as ER and grade combined, will facilitate inclusion of minimal pathology information in multifactorial modeling of individually rare variants.

Further, we provide, for the first time, LR estimates for TN status that can be applied when grade information is not recorded, with estimates associated with TN status comparable to those for ER-negative-grade 3 (for *BRCA1*) and ER-positive-grade 3 (for *BRCA2*). Altogether, these refined LRs will improve the clinical classification of *BRCA1* and *BRCA2* variants, particularly those identified in women with later age at diagnosis.

Our ER-grade analysis results for *BRCA1* are consistent with results from analysis of raw data for a smaller dataset of 600 *BRCA1* carriers aged younger than 60 years and 258 age-matched non-carriers from the Breast Cancer Linkage Consortium, which yielded LRs of 1.94 (95% CI = 1.05 to 3.56) and 2.95 (95% CI = 2.41 to 3.62) for ER-negative grade 2 and ER-negative grade 3 tumors, respectively [26],[27]. However, the current study demonstrates that ER-negative grade 2 or 3 status is more predictive of positive *BRCA1* status in women diagnosed at older than 50 years compared with younger than 50 (for example, for ER-negative-grade 3, LR ≥50 years is 4.13 (95% CI = 3.70 to 4.62) versus LR <50 years of 3.16 (95% CI = 2.96 to 3.37); *P*_*het*_ <0.0001. These observations reflect the fact that although the overall proportion of ER-negative high-grade tumors is lower for older onset (54.5%) than younger onset (67.1%) *BRCA1* carriers (as previously reported [33],[40]), the proportion of ER-negative high-grade tumors differs much more markedly for older-onset (12.8%) than younger-onset (20.8%) cases with no identified mutation in *BRCA1* or *BRCA2*.

In addition, not reported in previous smaller studies [6],[7],[41], our results show that ER-positive grade 2 or 3 status is a stronger *negative* predictor of *BRCA1* mutation status in women diagnosed before age 50 years compared with those diagnosed at age 50 years or older. These patterns reflect changes in the frequency of ER status and grade as a function of age in the non-carrier cases, rather than large changes in the frequency of these features in the carriers. Similarly, the findings for *BRCA2* are consistent with those from a previous study of 157 *BRCA2* mutation carriers and 314 mutation-negative familial breast cancer cases, which indicated that *BRCA2*-associated tumors were more likely to be ER-positive than were control tumors, when accounting for grade (OR, 2.09; 95% CI, 1.21 to 3.63; *P* =0.008) [13].

However, age-stratified analysis highlighted that ER-negative grade 3 tumor status modestly predicted positive *BRCA2* mutation status in women diagnosed at age 50 years or older, indicating that grade is a more important factor than ER status in predicting *BRCA2* tumors. We attempted to assess pathology difference by mutation type (missense versus truncating), an issue that has not previously been addressed rigorously because of the limited availability of pathology information for proven high-risk missense mutations. However, even in our very large dataset, the number of proven pathogenic missense mutations remained small, and it is apparent that future even larger studies will be needed to address this question.

The associations between *BRCA1* mutation status and TN phenotype are consistent with those observed for ER-negative, high-grade tumors. They are also consistent with prior evidence that *BRCA1* mutation carriers are enriched for the “basal” tumor phenotype that is highly concordant with TN status. A recent meta-analysis assessing the prevalence of *BRCA1* mutations in TN versus non-TN breast cancer patients from largely high-risk breast cancer populations [42] estimated a risk of 5.65 (95% CI, 4.15 to 7.69) based on analysis of 236 *BRCA1* mutation carriers and 2,297 non-carriers. In addition, these authors predicted that approximately two in nine women with TN breast cancer and additional high-risk features (early onset or family history) harbor a *BRCA1* mutation [42]. TN status has not been obviously linked to *BRCA2* mutation status previously; however, a recent study of 43 deleterious *BRCA1/2* mutation carriers identified from screening of 409 Chinese familial breast cancer cases reported that TN phenotype was more likely to be exhibited by both *BRCA1* (*P* =0.001, 69%, *n* = 16) and *BRCA2* (*P* =0.01, 46%, *n* = 27) carriers identified in their cohort, compared with non-carriers (23%; *n* = 366) [43]. In contrast, a similar study of 221 Korean familial breast cancer patients [44] identified 81 deleterious mutation carriers, and demonstrated increased TN phenotype for *BRCA1* mutation carriers (*P* <0.00001,57%, *n* = 35), but not *BRCA2* mutation carriers (*P* =0.9, 13.9%, *n* = 36) compared with non-carriers (13%, *n* = 130). Neither of these studies presented their findings for cases stratified by diagnosis age 50 years or older.

Our study has shown that TN phenotype is modestly predictive of *BRCA2* mutation status in cases diagnosed at 50 years or older, due to a lower TN frequency in non-carriers versus *BRCA2* mutation carriers in this age group. Reassuringly, these TN frequency differences mirror the results seen for ER-negative grade 3 status in non-carriers and *BRCA2* mutation carriers, an analysis based on a much larger sample set.

### Possible impact of study limitations

We acknowledge several limitations of our study. Ideally, our reference group would have been drawn from the same source as the mutation carriers, as there may be differences between non-BRCA familial cases and unselected cases. However, in the subset of 30,233 BCAC cases that had data on family history, we did not see any significant differences between this group and the remainder of the sample in terms of the pattern of histological features, nor with those who indicated no first- or second-degree relatives with breast cancer.

In our analyses, we are implicitly assuming that testing for *BRCA1/2* mutations was independent of the histopathology features used for prediction of mutation status. Although recently some features with therapeutic implications, such as TN status, are being used as a criterion for testing in some centers, we believe that the vast majority of our CIMBA carriers were tested solely on the basis of their family history. This analysis assumes that mutation testing in CIMBA sample sets was not directed by tumor histology. Mutation status was not known for all BCAC samples. However, mutation testing of BCAC samples had been performed for many studies with selected design that might be expected to be enriched for *BRCA1* and *BRCA2* mutation carriers, and these known mutation carriers were excluded from analysis.

Further, our sensitivity analyses suggest that, at very most, 2.5% of BCAC cases might carry an undetected mutation, and also show that our results would not be substantially affected by this level of contamination of the reference group.

The various sensitivity analyses conducted for the ER-grade dataset provided no convincing evidence for obvious differences for the factors being assessed. We did not see any marked difference in LR estimates for analyses restricted to individuals of European ancestry, but the small numbers of cases from other ethnic/racial groups did not allow us to assess reliably tumor histopathological features for other ethnic groups, and so may not be generalizable to patients of non-European ancestry. Although it is possible that variation in pathology grading and IHC testing methods might occur between countries or over time, our investigations provided no evidence that such differences would meaningfully confound interpretation of the results, and thus should not limit the use of the information generated for multifactorial likelihood analysis of *BRCA1* or *BRCA2* variants across continents.

### Use of revised LR estimates for future multifactorial likelihood analyses

**Figure 1:**
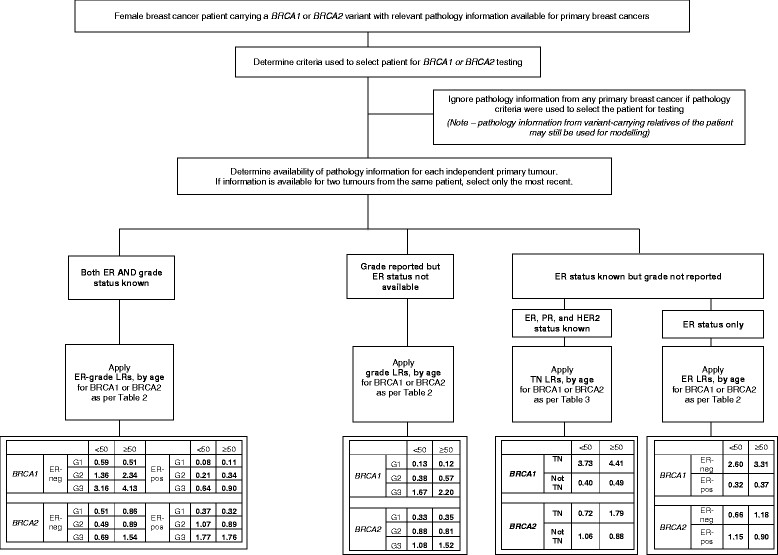
**Proposed strategy for application of pathology likelihood ratios in multifactorial likelihood analysis of** ***BRCA1*** **or** ***BRCA2*** **rare sequence variants.** Cases carrying a variant of uncertain clinical significance, and with information on relevant pathology variables, are first assessed to determine that breast tumor pathology information was not a criterion used to trigger gene testing. ER, estrogen-receptor breast tumor status; PR, progesterone-receptor breast tumor status; HER2, HER2 breast tumor status; TN, triple-negative breast tumor status; Not TN, breast tumor status not triple-negative, after measurement of ER, PR, and HER2 status; ER-neg, ER-negative status; ER-pos, ER-positive status; G, grade; <50, breast cancer diagnosis at younger than 50 years for tumor with relevant pathology data; ≥50, breast cancer diagnosis at 50 to 70 years for tumor with relevant pathology data.

This study has re-estimated the likelihood of *BRCA1* or *BRCA2* mutation status associated with breast tumor features commonly measured in the clinical setting, by analyzing much larger datasets than previously used for this purpose. Our findings provide measures of confidence in the individual LR estimates, and in particular, allow age at diagnosis to be incorporated into the pathology component of the multifactorial likelihood model. Figure 1 provides a flowchart indicating the proposed application of pathology-based LRs, dependent on what breast tumor pathology information is available for a variant carrier. As indicated, ER-grade LRs should be applied in preference to other pathology LR estimates, where both ER and grade information is available. The ER-grade LRs were derived from analysis of the largest sample sizes and thus have the greatest precision, and application of 12 strata provided by three grade categories refines both positive and negative prediction of mutation status. For example, a patient with a high-grade ER-negative tumor is three- to fourfold more likely to carry a *BRCA1* mutation than not, whereas a patient with a low-grade ER-positive tumor is about 10 times more likely to be mutation-negative than mutation-positive. Given that grade and ER are almost universally used to assess prognosis and predict response to antiestrogen therapies, these features are generally readily available on standard pathology reports.

This study could not provide a comparison to existing LR estimates of *BRCA1* mutation status based on ER-CK status, determined from analysis of 182 *BRCA1* and 109 age-matched cases [6]. However, we caution that very large confidence limits exist around the previously estimated LRs for ER-CK characteristics, and recommend further study of large carrier and reference sample sets to provide more-robust LR estimates for ER-CK phenotype in relation to mutation status.

It is important to note that the LRs estimated in this study were from analysis of sample sets that were, to our knowledge, unselected for tumor pathology status. Therefore, it will be necessary to consider potential for bias when individuals are screened for mutations on the basis of their tumor phenotype. This is expected to occur increasingly, now that *BRCA1/2* mutation-prediction programs such as BOADICEA include pathology as a component [16], and given recent evidence supporting implementation of the National Comprehensive Cancer Network (NCCN) guidelines that recommend testing of all TN breast cancer patients aged 60 years or younger [45]. In this scenario, multifactorial likelihood analysis should exclude tumor-pathology information from individuals who had previously contributed to risk prediction used to prioritize families for mutation screening. However, pathology data generated subsequently from other variant carrier relatives can still provide independent information toward variant classification.

We are aware that, in the future, other tumor characteristics could provide useful information for variant classification. Array Comparative Genomic Hybridization (CGH) has been shown as an effective method to identify *BRCA1*-mutated breast cancers and sporadic cases with a *BRCA1*-like profile [46],[47] for appropriate chemotherapeutics, and to distinguish *BRCA2*-mutated tumors from sporadic breast tumors [48]. If introduced widely as a routine test, this approach might be considered in the future as an alternative predictor in multifactorial modeling. Furthermore, the mutual exclusivity of *BRCA1*-germline mutations and *BRCA1* promoter methylation in tumors with *BRCA1*-like CGH profile [49] suggests that *BRCA1* promoter methylation tests would add value in distinguishing somatic from germline loss of *BRCA1* function, as is established for clinical testing triage and variant classification relating to *MLH1* mismatch repair cancer-predisposition gene [50].

Alternatively, genome-wide tumor-methylation profiles may prove of value to distinguish between individual with and without a germline *BRCA1* mutation [51]. Further, additional substratification of currently used histological features may add value in prediction of mutation status. Options include PTEN loss of expression in addition to TN status as a marker of *BRCA1* mutation status [11], or gene-expression arrays to identify *BRCA2* mutation carriers among the subset of luminal B tumors [15].

Recent research has also shown the value of considering further stratification of breast cancer subtype in the prediction of *BRCA* mutation status. For example although ER-negative status clearly predicts *BRCA1* mutation status, even ER-positive *BRCA1*-related breast cancers are more likely to be grade 3, CK14+, and show high mitotic rate compared with ER-positive sporadic cancers [52].

In addition, possibilities exist to extend histopathological analyses to tumors other than female breast cancer. The combination of modified Nottingham grade 3 serous or undifferentiated histology, prominent intraepithelial lymphocytes, marked nuclear atypia with giant nuclei, and high mitotic index has recently been reported to be a significant predictor of *BRCA1* mutation status in women with epithelial ovarian cancer [53]. Further, breast tumors of male *BRCA2* mutation carriers are more likely to present as high-grade, PR-negative, and relatively high rates of HER2-positivity with a micropapillary component to histology have been reported [54],[55]. Investigation of these features in larger sample sizes should be considered in the future.

Although this article has focused on the utility of histopathologic features of breast cancers in the context of the classification of variants in the *BRCA1* and *BRCA2* genes, these results should also be useful in a range of other applications. The information provided in the main tables can be used to estimate sensitivities and specificities of histopathological predictors by broad age-group (for example, triple-negative tumor status has sensitivity of 0.67 and specificity 0.82 for detection of *BRCA1* mutation status in women diagnosed at younger than age 50 years, whereas the sensitivity is 0.57 and the specificity 0.87 for women diagnosed at age 50 or older. As such, these results, in conjunction with other predictors of mutation status, could be useful to guide systematic genetic testing of germline DNA from patients to determine the appropriateness of the use of PARP inhibitors in therapy. The results arising from this study are also likely to inform future development of parallel models, which estimate the probability of an individual carrying a *BRCA1* or *BRCA2* mutation, to determine eligibility and/or priority for genetic testing (in particular, the BOADICEA model, which has recently been updated to include additional histopathologic characteristics from large data resources [56]).

## Conclusions

The results from this large-scale analysis refine likelihood ratio estimates for predicting *BRCA1* and *BRCA2* mutation status by using commonly measured histopathological features. We demonstrate the importance of considering age at diagnosis for analyses, and show that grade is more informative than ER status for *BRCA2* mutation-carrier prediction. The estimates will improve *BRCA1* and *BRCA2* variant classification by using multifactorial likelihood analysis, and inform patient mutation testing and clinical management.

## Additional file

## Electronic supplementary material

Additional file 1: Table S1.: CIMBA and BCAC sample sets included in analysis.* **Table S2.** Unstratified estimated likelihood ratios (LRs) for predicting *BRCA1* or *BRCA2* mutation status defined by breast tumor ER-grade phenotype. **Table S3.** Unstratified estimated LRs for predicting *BRCA1* or *BRCA2* mutation status defined by breast tumor triple-negative phenotype. **Table S4.** Unstratified estimated LRs for predicting *BRCA1* or *BRCA2* mutation status defined by breast tumor TN-grade phenotype.* (XLSX 34 KB)

Below are the links to the authors’ original submitted files for images.

Authors’ original file for figure 1

## Abbreviations

BCAC: Breast Cancer Association Consortium
CGH: Array Comparative Genomic Hybridization
CIMBA: Consortium of Investigators of Modifiers of BRCA1/2
CK14: cytokeratin 14
CK17: cytokeratin 17
CK5/6: cytokeratin 5/6
EGFR: epidermal growth factor receptor
ENIGMA: Evidence-based Network for the Interpretation of Germline Mutant Alleles
ER: estrogen receptor
HER2: Human epidermal growth factor receptor 2
IHC: immunohistochemical
LR: likelihood ratio
NCCN: National Comprehensive Cancer Network
PR: progesterone receptor
PTEN: phosphatase and tensin homolog
TN: triple negative
