# Supplementary figures and images for: Refined histopathological predictors of BRCA1 and BRCA2mutation status: a large-scale analysis of breast cancer characteristics from the BCAC, CIMBA, and ENIGMA consortia
Source: Breast Cancer Res. 2014 Dec 23;16:3419. doi: 10.1186/s13058-014-0474-y (PMC4352262; doi:10.1186/s13058-014-0474-y)

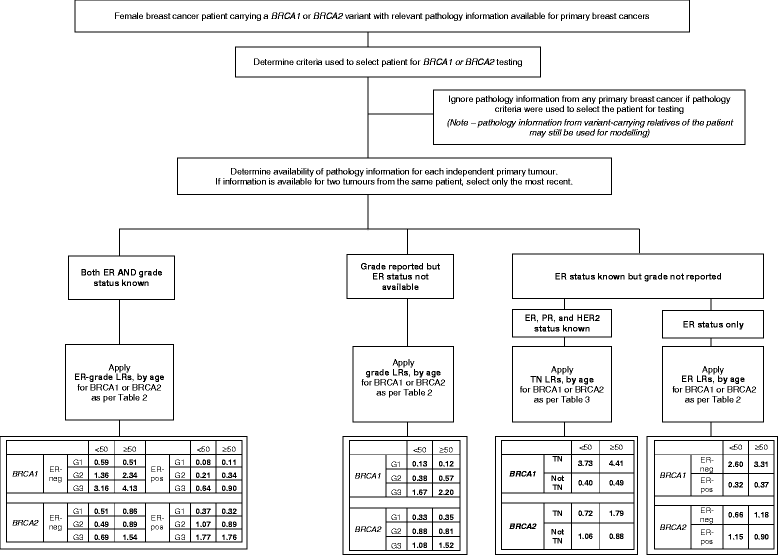

Supplement: Supplementary file 2 — Authors’ original file for figure 1 [file 13058_2014_474_MOESM2_ESM.gif]
